# A *Francisella* Mutant in Lipid A Carbohydrate Modification Elicits Protective Immunity

**DOI:** 10.1371/journal.ppat.0040024

**Published:** 2008-02-08

**Authors:** Duangjit Kanistanon, Adeline M Hajjar, Mark R Pelletier, Larry A Gallagher, Thomas Kalhorn, Scott A Shaffer, David R Goodlett, Laurence Rohmer, Mitchell J Brittnacher, Shawn J Skerrett, Robert K Ernst

**Affiliations:** 1 Department of Medicine, University of Washington, Seattle, Washington, United States of America; 2 Department of Immunology, Faculty of Medicine Siriraj Hospital, Mahidol University, Bangkok, Thailand; 3 Department of Immunology, University of Washington, Seattle, Washington, United States of America; 4 Department of Genome Sciences, University of Washington, Seattle, Washington, United States of America; 5 Department of Medicinal Chemistry, University of Washington, Seattle, Washington, United States of America; Stanford University School of Medicine, United States of America

## Abstract

Francisella tularensis (Ft) is a highly infectious Gram-negative bacterium and the causative agent of the human disease tularemia. Ft is designated a class A select agent by the Centers for Disease Control and Prevention. Human clinical isolates of Ft produce lipid A of similar structure to Ft subspecies *novicida* (Fn), a pathogen of mice. We identified three enzymes required for Fn lipid A carbohydrate modifications, specifically the presence of mannose (*flmF*1), galactosamine (*flmF*2), or both carbohydrates (*flmK*). Mutants lacking either galactosamine (*flmF*2) or galactosamine/mannose (*flmK*) addition to their lipid A were attenuated in mice by both pulmonary and subcutaneous routes of infection. In addition, aerosolization of the mutants (*flmF*2 and *flmK*) provided protection against challenge with wild-type (WT) Fn, whereas subcutaneous administration of only the *flmK* mutant provided protection from challenge with WT Fn. Furthermore, infection of an alveolar macrophage cell line by the *flmK* mutant induced higher levels of tumor necrosis factor-α (TNF-α) and macrophage inhibitory protein-2 (MIP-2) when compared to infection with WT Fn. Bone marrow–derived macrophages (BMMø) from Toll-like receptor 4 (TLR4) and TLR2/4 knockout mice infected with the *flmK* mutant also produced significantly higher amounts of interleukin-6 (IL-6) and MIP-2 than BMMø infected with WT Fn. However, production of IL-6 and MIP-2 was undetectable in BMMø from MyD88^−/−^ mice infected with either strain. MyD88^−/−^ mice were also susceptible to *flmK* mutant infection. We hypothesize that the ability of the *flmK* mutant to activate pro-inflammatory cytokine/chemokine production and innate immune responses mediated by the MyD88 signaling pathway may be responsible for its attenuation, leading to the induction of protective immunity by this mutant.

## Author Summary

Bacterial pathogens modify outer membrane components, such as lipid A or endotoxin, the lipid anchor of lipopolysaccharide, to enhance the ability to colonize, spread to different tissues, and/or avoid the host's immune defenses. Lipopolysaccharide also plays an essential role in maintaining membrane integrity and is a key factor in host innate immune recognition of Gram-negative bacterial infections. Francisella tularensis is the causative agent of the human disease tularemia and is classified as a category A select agent. Francisella novicida (Fn) is the murine counterpart of F. tularensis. The structure of Francisella spp. lipid A is unique in that it is modified by various carbohydrates that play a role in virulence and altered endotoxicity. In our study, we identified and defined the role of three genes involved in the carbohydrate modification of the base Fn lipid A structure. We showed that the lack of specific modification(s) of the Fn lipid A molecule lead to bacterial attenuation and activation of a protective immune response against a lethal wild-type infection. Therefore, alteration of Francisella lipid A structure may represent a pathogenesis strategy common to the *Francisella* species, and specific lipid A mutant strains may be candidates for inclusion in future vaccine studies.

## Introduction

Francisella tularensis (Ft) is a Gram-negative intracellular bacterium that causes the severe and often fatal disease tularemia in humans. Infection can occur through skin contact, insect bite, or inhalation of contaminated air. Ft is classified as a category A bioterrorism agent due to its high infectivity and mortality, transmission by an airborne route of infection [1–3], and development as a bioweapon. Francisella is categorized into numerous subspecies: *tularensis* (Type A), *holarctica* (Type B), *mediasiatica*, and *novicida*. Ft Type A and Type B cause disease in humans, with Type A being the most virulent. Francisella novicida (Fn) causes a severe disease in a murine model but is not virulent in immunocompetent humans. Interestingly, all subspecies share greater than 95% DNA sequence homology, suggesting a close genetic relationship and allowing Fn to be considered an acceptable model for studying *Francisella* LPS biosynthesis and pathogenicity [1,4].

Lipid A, the biologically active component of Gram-negative bacterial lipopolysaccharide (LPS), is responsible for various pathological responses in Gram-negative bacterial infections [5–7]. Classical biphosphorylated, hexa-acylated lipid A species from Escherichia coli can activate pro-inflammatory responses through Toll-like receptor 4 (TLR4), while tetra- or penta-acylated lipid A species have significantly diminished immunostimulatory activity [5,8]. The lipid A molecule can be modified by the addition of various carbohydrates, removal of phosphate moieties, or variation in the length and/or order of fatty acid chains, altering recognition by the host innate immune system. *Francisella* LPS and lipid A molecules lack immunostimulatory activity and are not recognized by TLR2 or TLR4 [9,10] and display little to no endotoxic properties in galactosamine-treated mice, by limulus assay (a standard for determining LPS endotoxin potential), after aerosolization in mice, or by stimulation of mononuclear cells to release cytokines [11–13].

**Figure 1:**
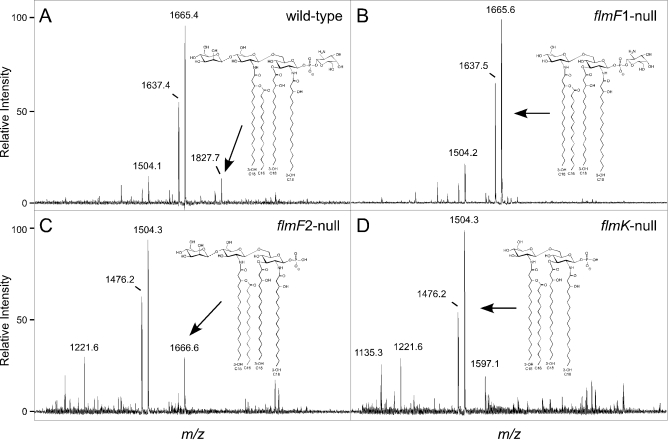
Lipid A Structures and Profiles from MALDI-TOF Mass Spectrometry Analysis of *Francisella* (A) WT, (B) *flmF*1 Mutant, (C) *flmF*2 Mutant, and (D) *flmK* Mutant

The β-(1,6)-linked diglucosamine backbone structure of *Francisella* lipid A has amide-linked fatty acids at the 2 ((18:0)-3-OH) and 2′ positions and ester-linked fatty acids at the 3 ((18:0)-3-OH), but not the 3′ positions [14–17]. A fatty acid (16:0) is attached to the 2′ fatty acid, forming an acyloxyacyl group ((18:0)-3-(16:0)). *Francisella* subspecies lipid A has a single phosphate moiety at the 1 position of the reducing glucosamine residue. The phosphate is further substituted with the positively charged sugar galactosamine [15], which was recently shown to be α-linked in Fn by Wang et al. [18] (Figure 1B, *m/z* 1665)*.* Finally, the 4′ phosphate is absent and can be removed by the recently identified phosphatase LpxF [18].

In this study, we screened transposon-generated Fn mutants for enzymes involved in lipid A biosynthesis and modification. Two proposed dolichyl phosphate-mannose synthase-like enzymes, FlmF1 (FTN_1403) and FlmF2 (FTN_0545), required for the synthesis of the undecaprenyl-phospho-mannose or galactosamine donor lipid, respectively, and a proposed glycosyltransferase enzyme, FlmK (FTN_0546), required for the addition of both mannose and galactosamine to lipid A, were identified.

Fn mutants (*flmF*2 and *flmK*) were attenuated in mice after subcutaneous and pulmonary infection and provided protection against a lethal wild-type (WT) Fn infection. In addition, the *flmK* mutant stimulated an increased innate immune response as compared to WT Fn or other bacterial lipid A biosynthetic mutants. This increased immune activation appears to involve the MyD88 signaling pathway. This study shows attenuation of virulence and the induction of protective immunity in both subcutaneous and pulmonary routes of infection by Fn mutants with altered lipid A.

## Results

### Enzymes Involved in Carbohydrate Modification of *Francisella* Lipid A

In enteric bacteria, two enzymes are required for the synthesis of the undecaprenyl-phospho-aminoarabinose donor lipid (PmrF/ArnC) and addition (PmrK/ArnT) of aminoarabinose to lipid A [19,20]. The genes encoding these enzymes in Fn were determined using clusters of orthologous groups (COG), gene ontology (GO), and/or PFAM database–based searches for conserved motifs to the Salmonella typhimurium enzymes. Twenty-three putative Fn orthologs of the S. typhimurium PmrK and PmrF genes were identified. Lipid A, derived from individual transposon mutants in the Fn orthologs after growth at 37 °C using an ammonium hydroxide/isobutyric acid extraction method, was subjected to matrix-assisted laser desorption ionization time-of-flight (MALDI-TOF) mass spectrometry (MS) analysis in the negative ion mode. Only three individual Fn mutants were identified that showed altered lipid A carbohydrate modification, as compared to WT Fn (Figure 1).

Negative ion MALDI-TOF MS analysis of lipid A isolated from the WT Fn strain (U112) after growth at 37 °C showed a dominant peak at mass/charge (*m/z*) 1665 that corresponded to a tetra-acylated base structure with a galactosamine residue at the 1 position phosphate (Figure 1A). A minor ion species at *m/z* 1504 corresponded to the loss of the galactosamine modification (-Δ161 *m/z*), whereas the ion species at *m/z* 1827 represented the addition of a second hexose residue (+Δ162 *m/z*). Finally, the ion peak at *m/z* 1637 consisted of lipid A structures with smaller acyl chains compared to *m/z* 1665 and was the dominant ion in lipid A isolated after growth at 25 °C [14].

Fn mutant 1, with a transposon inserted in the FTN_1403 gene, (http://www.francisella.org/) lacked the minor ion species at *m/z* 1827, as compared to WT Fn spectra (compare Figure 1A and 1B), suggesting the loss of a single hexose moiety (−Δ162 *m/z*) from the Fn lipid A structure. This Fn gene was shown to be a homolog of *S. typhimurium pmrF* (Figure S1A and S1B) and was named Fn *flmF*1 (*Francisella* lipid A modification). Due to the presence of galactosamine in the major lipid A structure at the 1 position (*m/z* 1665), we propose that this second hexose residue is attached directly to the glucosamine backbone at the 4′ position. To determine the identity of the unknown hexose sugar present in WT Fn, but not *flmF*1, lipid A samples isolated after growth at 37 °C were hydrolyzed to obtain individual carbohydrate residues and analyzed as the trimethylsilyl derivatives using gas chromatography-mass spectrometry (GC-MS) analysis. GC-MS electron-impact mass spectra indicated that the additional hexose was mannose (unpublished data).

The presence of the phosphate-free addition of mannose found in this study was novel to *Francisella* lipid A biosynthesis, though it had been previously described in a variety of purple sulfur phototrophic bacteria [21–23] and the obligate predatory bacterium *Bdellovibrio* [24]. The significance of this modification in Fn is currently unknown, however upon growth at low temperatures (25 °C or lower), increased levels of mannose were observed [14]. Interestingly, the presence of mannose was observed only in lipid A isolated from either Type A F. tularensis subspecies *tularensis* (five of seven isolates) [10] or Fn strain U112, but not Type B F. tularensis subspecies *holarctica* (zero of 11 isolates), or F. tularensis subspecies *mediasiatica* (zero of three isolates) after growth at 25 °C (unpublished data).

Fn mutant 2, with a transposon inserted in the FTN_0545 gene, showed a major peak at *m/z* 1504, which represented the parent tetra-acylated lipid A structure lacking the 1 position phosphogalactosamine (−Δ*m/z* 161) residue (Figure 1C). The ion species at *m/z* 1666 represented a tetra-acylated structure that contained mannose but lacked galactosamine (*m/z* 1504 ion + 162 mass units). This gene was shown to be a second homolog of *S. typhimurium pmrF* (Figure S1A and S1B) and was named Fn *flmF*2. Interestingly, lipid A isolated from the Type B strain, LVS (Live Vaccine Strain), does not contain galactosamine, suggesting one possible mechanism for the avirulence/protection phenotype of this strain.

Finally, Fn mutant 3, with a transposon insertion in the FTN_0546 gene, showed a major ion peak at *m/z* 1504, which corresponded to a lipid A molecule that did not contain either carbohydrate modification (loss of *Δm/z* 161 (galactosamine) and 162 (mannose) residues) (Figure 1D). Therefore, this gene functions to transfer two sugar residues to the lipid A backbone structure. This gene was shown to be a homolog of *S. typhimurium pmrK* (Figure S1A and S1C) and was named Fn *flmK*.

The absence of specific carbohydrate modifications was confirmed by gas chromatography/mass spectrometry analysis for all Fn mutant strains (unpublished data). The individual Fn lipid A biosynthetic mutants did not affect O-antigen production. WT O-antigen laddering profiles, as determined by tricine SDS-PAGE gel electrophoresis using whole cell preparations or purified LPS, were observed for all strains tested (unpublished data).

### Attenuation of F. novicida Lipid A Mutants in Infected Mice

**Figure 2:**
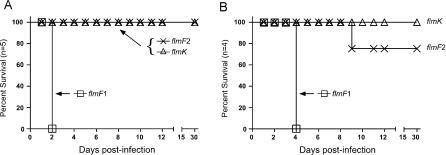
Mouse Survival after F. novicida Infection (A) C57BL/6 mice (*n* = 5) were injected subcutaneously with *flmF*1 (1,360 cfu), *flmF*2 (960 cfu), or *flmK* (1,280 cfu). Mice infected with lethal dose of WT Fn died at day 2 post-infection (unpublished data), (B) C57BL/6 mice (*n* = 4) were exposed to aerosolized *flmF*1 (deposition = 66 cfu/lung), *flmF*2 (120 cfu/lung), or *flmK* (71 cfu/lung). Mice infected with lethal dose of WT Fn died at day 4 post-infection (unpublished data). Representative of two independent experiments.

Using murine models of infection (subcutaneous and pulmonary), we determined the role of the individual lipid A biosynthetic mutants in virulence. Initially, C57BL/6 and BALB/c mice were infected subcutaneously to mimic zoonotic transmission, with either WT Fn (LD_100_ ∼ 1–10 cfu) or the three individual mutants, and their disease symptoms were observed. The *flmF*2 and *flmK* mutants were attenuated in mice, as all of the infected mice survived infection, in contrast to those infected with WT Fn or the *flmF*1 mutant (Figure 2A), which died 2 d post-infection. Similar results for all strains were observed in BALB/c mice (unpublished data).

To mimic the air-borne route of infection, C57BL/6 mice were infected with WT Fn or the mutants using a nose-only chamber to ensure that only an airway infection was achieved. It was previously determined that mice infected with as few as 5 cfu/lung via exposure to aerosolized WT Fn died on day 4 post-infection (S. J. Skerrett, unpublished data). C57BL/6 mice were exposed to aerosolized *flmF*1, *flmF*2, or *flmK* mutant bacteria (resulting in depositions of 66–120 cfu/lung). Similar to the subcutaneous infection studies, the *flmK* mutant was attenuated and mice infected with this mutant showed no signs of illness and uniformly survived the infection (Figure 2B). However, mice exposed to the *flmF*2 mutant showed mild clinical manifestations of disease (scruffy coat, lethargy) with a single mouse dying on day 9. In contrast to the *flmF*2 and *flmK* mutants, the *flmF*1 mutant retained its virulence and all *flmF*1-infected mice died at day 4, kinetics similar to infection with WT bacteria. This finding was similar to the results observed in mice infected via the subcutaneous route. This avirulent phenotype for the *flmF*2 and *flmK* mutants was not due to attenuation of growth in vitro, as the mutant strains displayed similar doubling times to WT Fn when grown in a rich culture medium (Figure S2A). In addition, similar numbers of bacteria were recovered from cultured alveolar macrophages (MH-S cell line) after infection with both the mutants and WT Fn (Figure S2B).

### Bacterial Replication in Infected Mice

**Figure 3:**
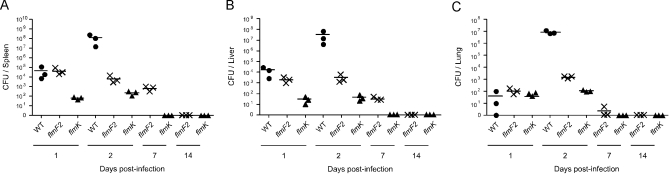
Bacterial Burden in (A) Spleen, (B) Liver, and (C) Lung of Mice after Subcutaneous Infection with WT, *flmF*2, or *flmK* Mutant Bacteria C57BL/6 mice (*n* = 3 each group) were injected with WT (•, 520 cfu), *flmF*2 (×, 465 cfu), or *flmK* (▴, 425 cfu) mutant bacteria. Mice infected with WT Fn were all dead by day 2 post-infection. Representative of two independent experiments.

To determine the kinetics of bacterial replication and spread following infection, C57BL/6 mice were infected subcutaneously with WT or *flmF*1, *flmF*2, or *flmK* mutant bacteria (∼400–500 cfu). Spleens, livers, and lungs were harvested from infected mice at day 1, 2, 4, 7, and 14 post-infection and plated to determine individual organ burden. WT and *flmF*1 bacteria were able to efficiently replicate in vivo: ∼10^4^ and 10^8^ cfu were recovered from the spleen and liver within 1 and 2 d post-infection, respectively (Figure 3 and unpublished data). In contrast, lower numbers of *flmK* mutant bacteria (∼10–100 cfu) were found in the spleen and liver at 1 d post-infection. The *flmK* mutant was completely cleared by day 4 post-infection, as bacteria were not found in any organ examined. The *flmF*2 mutant initially replicated, with bacterial deposition in the spleen increasing to ∼10^4^ cfu at day 1 post-infection, but it was eventually cleared by day 14 (Figure 3). These results show that mice were able to clear the attenuated *flmK* and *flmF*2 mutants but not the virulent *flmF*1 or WT bacteria.

**Figure 4:**
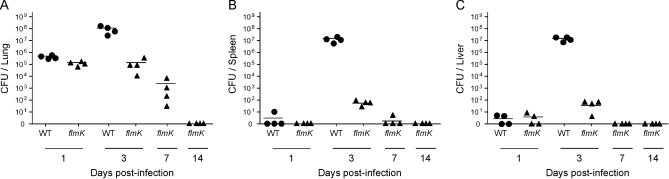
Bacterial Burden in (A) Lung, (B) Spleen, and (C) Liver after Exposure of C57BL/6 Mice to Aerosolized WT Fn (•, deposition = 100 ± 29 cfu/lung) or *flmK* mutant (▴, deposition = 146 ± 62 cfu/lung) Mice infected with WT Fn were all dead by day 4 post-infection. Representative of two independent experiments.

Similar results were observed in C57BL/6 mice infected by aerosolization of WT or *flmK* mutant bacteria (∼100 cfu/lung). Initially, both the WT and *flmK* bacteria replicated in the lungs, reaching about 3-log of the initial dose at day 1 post-infection. However, by day 3 post-infection, higher bacterial burdens were observed in the lungs, spleens, and livers of the WT-infected animals as compared to those infected with the *flmK* mutant (Figure 4). The bacterial burden in *flmK*-infected mice was cleared by day 14 post-infection.

### Innate Immune Response to *flmK* Mutant

**Figure 5:**
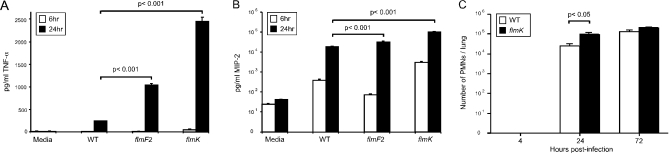
Pro-Inflammatory Cytokine Production by Infected Murine Macrophages Mean ± SEM of (A) TNF-α and (B) MIP-2 concentration from MH-S culture supernatants after infection with WT, *flmF*2, or *flmK* mutant bacteria (MOI ∼100) at 6 h (open bars) and 24 h (filled bars) post-infection. Representative of two independent experiments. Differences were not significant at 6 h post-infection. (C) PMN enumerations in BAL fluid of mice infected with aerosolized WT (open bar) or *flmK* mutant (filled bar) bacteria. Representative of two independent experiments. There was no significant difference at 4 and 72 h post-infection.

Since both the *flmK* and *flmF*2 mutant strains were attenuated in the murine model via both the pulmonary and subcutaneous routes of infection, we determined the potential role of the host innate immune system in recognition and clearance of these mutants. To test for enhanced recognition and/or increased pro-inflammatory cytokine and chemokine production, a mouse alveolar macrophage cell line (MH-S cells) was infected with WT or mutant bacteria. Culture supernatants were collected at 6 and 24 h post-infection and assayed for TNF-α and MIP-2 production by ELISA. At 24 h post-infection, the *flmK* and *flmF*2 mutant stimulated enhanced TNF-α (Figure 5A) and MIP-2 (Figure 5B) production relative to WT Fn (*p* < 0.001). To determine the inflammatory response during pulmonary infection, polymorphonuclear leukocytes (PMN) in bronchoalveolar lavage (BAL) fluids from mice exposed to aerosolized WT Fn or *flmK* mutant bacteria were enumerated. The number of PMN in BAL fluids was significantly higher at 24 h post-inhalation of the *flmK* mutant as compared to WT Fn (Figure 5C).

**Figure 6:**
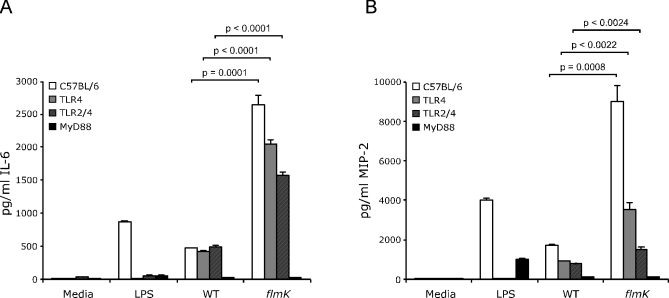
Cytokine and Chemokine Production by BMMø Mean ± SEM of (A) IL-6 and (B) MIP-2 concentration in culture supernatants at 24 h after infection of BMMø from C57BL/6 (open bars) or MyD88^−/−^ (filled bars) mice with WT or *flmK* mutant bacteria (MOI 100). Significant differences were observed between C57BL/6 and MyD88^−/−^ mice.

To further dissect the role of the innate immune response to the *flmK* mutant, the importance of the TLR system (and/or IL-1/IL-18) in controlling infection was tested in MyD88 knockout mice. Bone marrow–derived macrophage (BMMø) from C57BL/6 mice secreted significantly higher levels of IL-6 and MIP-2 in response to infection by the *flmK* mutant than in response to WT Fn (Figure 6A and 6B, MOI = 100, *p* < 0.001). Significant differences in the production of IL-6 (*p* < 0.02) and MIP-2 (*p* < 0.05) were also observed at MOI 10 (unpublished data). Levels of lactate dehydrogenase (LDH) in supernatants, harvested at 6 and 24 h after infection, from C57BL/6 BMMø infected with either strain of bacteria, at both MOI 10 and MOI 100 were similar (unpublished data). These results suggest that decreased production of both IL-6 and MIP-2 for WT-infected cells, as compared to the *flmK* mutant was not a result of cell death. Similar results were found in BMMø derived from TLR4^−/−^ and TLR2/4^−/−^ mice. However, IL-6 and MIP-2 were undetectable in MyD88^−/−^-derived BMMø cultures stimulated with either strain of Fn, demonstrating the requirement for MyD88 signaling in the innate immune response to *flmK* infection.

**Figure 7:**
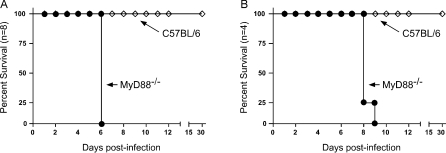
MyD88^−/−^ Mice Are Susceptible to *flmK* Mutant Infection MyD88^−/−^ (•) or C57BL/6 (⋄) mice were (A) exposed to aerosolized (30 cfu) or (B) infected subcutaneously (508 cfu) with *flmK* mutant bacteria. Representative of two independent experiments.

The importance of the MyD88 signaling pathway in the response to *flmK* mutant infection was further evaluated in vivo. MyD88^−/−^ mice and WT mice were exposed to aerosolized *flmK* bacteria (∼100 cfu) and disease development was monitored. MyD88^−/−^ mice were highly susceptible to *flmK* mutant infection and all died by day 6 post-infection, whereas all WT mice infected with *flmK* bacteria showed no signs of disease and survived for at least 30 d post-infection (Figure 7A). Similar results (Figure 7B) were observed after subcutaneous infection of MyD88^−/−^ mice (∼500 cfu of *flmK* mutant). Interestingly, all *flmK*-infected MyD88^−/−^ mice died by day 9 post-infection, a time point greatly delayed relative to WT mice infected with WT Fn.

One arm of the innate immune system is the eradication of colonizing microorganisms by nonspecific killing mechanisms. Antimicrobial peptides target the bacterial membrane via electrostatic interactions, leading to the disruption of the outer membrane. Therefore, we determined the susceptibility of the various lipid A mutants to polymyxin B, a cyclic cationic antimicrobial peptide, using a disc diffusion assay. Both the WT and the lipid A modification gene mutant bacteria (*flmF*1, *flmF*2, and *flmK*) were shown to be highly resistant to killing, whereas as a control a Fn lipid A biosynthetic 4′ position phosphatase-null mutant (*lpxF*) was susceptible, as previously shown [18] (Figure S3).

### Protection against a Lethal WT F. novicida Challenge

**Figure 8:**
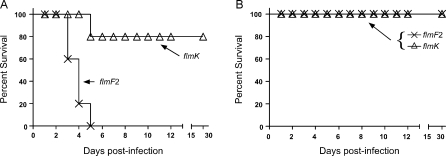
Mouse Survival after a Lethal WT F. novicida Challenge (A) C57BL/6 mice that survived primary *flmF*2 (×) or *flmK* (▵) subcutaneous infection were challenged with WT Fn (660 cfu) via the subcutaneous route. (B) C57BL/6 mice that survived primary *flmF*2 (×) or *flmK* (▵) pulmonary infection were challenged via the pulmonary route with WT Fn (25 cfu). Representative of two independent experiments.

To determine if mice that survived initial subcutaneous infection were protected from subsequent challenge with WT Fn, the surviving *flmF2* and *flmK* mice (∼30–35 d post initial infection) were challenged with a lethal dose of WT Fn (660 cfu). Mice that were initially infected with the *flmK* mutant acquired protective immunity (Figure 8A), as only a single mouse (*n* = 5) died on day 5 post challenge. As a control, naïve C57BL/6 mice were infected with WT Fn at the same dose and all died at day 2 post-infection (unpublished data). In contrast, mice initially infected with the *flmF*2 mutant were not protected against WT Fn infection, as all mice died by day 5 post WT challenge. Similar results were observed for both mutants in BALB/c mice that were initially infected subcutaneously (unpublished data).

Protection studies were also performed using mice that were initially infected by aerosolization. Using a lethal dose (25 cfu) of WT Fn for challenge, complete protection was obtained when mice were initially infected with either the *flmF*2 or *flmK* mutants (Figure 8B). As a control, naïve C57BL/6 mice were infected with WT Fn at the same dose and all died at approximately day 4 post-infection. These results show that initial infection/immunization using the *flmK* mutant provided nearly complete protection against WT Fn challenge via both pulmonary and subcutaneous routes of infection. Interestingly, the *flmF*2 mutant provided protection only through aerosolization but not through subcutaneous infection, suggesting that the route of initial infection may be important in the generation of a protective response.

## Discussion

Lipid A of *Francisella* is different from those of classical enteric bacteria in terms of its structure and biological activities. *Francisella* lipid A has tetra-acyl chains of 16–18 carbons in length and the phosphate moiety at the 4′ position is removed by the LpxF phosphatase enzyme. This molecule can be further modified by the addition of galactosamine onto the 1-phosphate moiety. Another unique modification is the presence of glucose at the 6′ position to free lipid A [25], which has a similar molecular mass as Fn lipid A that contains the mannose modification described above.

In this study, three genes required for the carbohydrate modification of Fn lipid A were identified. Two of the genes, FTN_1403 (*flmF*1) and FTN_0545 (*flmF*2), functioned similarly to the S. typhimurium PmrF enzyme and are proposed to be involved in the transfer of mannose or galactosamine, respectively, to undecaprenyl phosphate, a polyisoprenoid carrier lipid required for the transport of water soluble precursors across a lipid membrane (Figure 1A). Inactivation of the third gene, FTN_0546 (*flmK*), recently described by Wang et al. [18] for the addition of only galactosamine, resulted in the loss of both galactosamine and mannose modifications, suggesting that this gene functions to transfer the individual carbohydrate moieties from undecaprenyl phosphate to lipid A, similar to the function of PmrK in S. typhimurium. All three genes are highly homologous among the sequenced *Francisella* subspecies genomes (Type A – Schu4, WY96–3418; Type B – OSU18, LVS; Fn – U112) with overall amino acid sequence similarity of 98.3%–99.7%, suggesting a conserved function for these enzymes (Figure S1A).

Mutants in *flmF*2 and *flmK* were attenuated in mice by both pulmonary and subcutaneous routes of infection. Both mutant strains provided protection against a lethal WT *Francisella* infection and induced protective immunity in mice vaccinated via the pulmonary route of infection. Interestingly, only the *flmK* mutant provided protective immunity when initial infection was performed via subcutaneous injection.

As the *flmK* mutant showed the most promising outcome in terms of attenuation and induction of protective immunity, further work was focused on this mutant. We hypothesized that the *flmK* mutant would stimulate an increased immune response, particularly via the innate arm of host immunity. This hypothesis was supported by the findings that *flmK* mutant infection resulted in enhanced induction of pro-inflammatory cytokine and chemokine production in a mouse alveolar macrophage cell line and in BMMø in vitro and enhanced elicitation of neutrophils to the lungs in vivo, in comparison with WT Fn infection. We also showed that MyD88-mediated signaling was involved in the innate immune response stimulated by the *flmK* mutant, as the augmented cytokine response of BMMø to this mutant required MyD88, and infection with the *flmK* mutant was lethal in mice lacking MyD88 (albeit with delayed kinetics in comparison to the lethality of WT Fn infection in C57BL/6 mice). These observations suggested that the *flmK* mutant might be more readily recognized by TLRs than is WT type Fn. Indeed, we found that the augmented cytokine response of BMMø to the *flmK* mutant was partially dependent on TLR2 and TLR4. However, whereas MyD88-deficient mice succumbed to infection with the *flmK* mutant (Figure 7), no mortality was observed after subcutaneous *flmK* infection of mice lacking TLR4 or both TLR2 and TLR4 (unpublished data). Thus, MyD88-dependent responses that are independent of TLR2 and TLR4 appear to be essential to the protective response elicited by the *flmK* mutant. The significance of the MyD88 signaling pathway has been shown in a model of LVS infection, where susceptibility of MyD88 knock out mice to *Francisella* infection was enhanced, even at a very low dose of bacteria (10^6^-fold less than LD_50_) [26].

The importance of the innate immune response in control of Fn infection has been previously demonstrated. Recently, Wang et al. showed that a Fn mutant with a defect in LpxF, an enzyme that removes the phosphate moiety from the 4′ position of the lipid A molecule, was highly attenuated in vivo (intradermal injection into mouse footpad) [27], though it displayed a significant defect in growth in vitro in rich culture medium (TSBC). This mutant strain elicited increased cytokine responses and inflammatory cell recruitment as compared to WT Fn after intraperitoneal injection of mice. The LpxF-null strain was also more sensitive to the cyclic cationic antimicrobial peptide polymyxin B.

The ability of the *flmK* mutant to activate the innate immune response may be responsible for the rapid clearance of the bacteria from mice before causing disease symptoms. Furthermore, this recognition may also lead to the potent induction of adaptive immune responses that provide protection against WT *Francisella* challenge. Due to the close genetic relatedness and similarity in lipid A structure among all four subspecies of *Francisella* [10], it is likely that inactivation of the *flmK* gene in other subspecies would also result in bacterial attenuation and activation of adaptive immunity. Protection against WT bacterial challenge was obtained after initial priming with the mutants, especially with *flmK*. Activation of the innate immune system and the induction of a pro-inflammatory response via the MyD88-mediated signaling pathway were shown to be essential for clearance of *flmK* and possibly for protection. These results suggest that carbohydrate modifications of lipid A play an important role in bacterial virulence and protective immunity and may aid in the development of an effective *Francisella* vaccine in the future.

## Materials and Methods

### Bacterial strains.

WT Fn U112 was obtained from Francis Nano (University of Victoria, Victoria, Canada) and the U112 *lpxF* mutant (XWK4) was obtained from Christian Raetz (Duke University, Durham, North Carolina). A mutant library (http://www.francisella.org/) was generated by transposon random mutagenesis of Fn strain U112 as part of a screen for essential genes in Fn [28]. Bacteria were cultured overnight in tryptic soy broth medium supplemented with 0.1% cysteine (TSBC) at 37 °C [10].

###  Mouse strains.

C57BL/6 and BALB/c mice (female, 6–8 wk old) were purchased from Jackson Laboratories. Mice were maintained under specific pathogen-free conditions. Mice were 6–12 wk of age at the time of experimental infection. All animal experiments were approved by the Institutional Animal Care and Use Committee of the University of Washington (Seattle, Washington). TLR4^−/−^ and TLR2/4^−/−^ mice were kindly provided by Dr. C. B. Wilson (University of Washington, Seattle, Washington). MyD88^−/−^ mice were kindly provided by Dr. S. Akira (Osaka, Japan) [29]. They were backcrossed to C57BL/6 mice for eight generations and then intercrossed to generate MyD88^−/−^ mice [30].

### Screening for mutants with a defect in lipid A synthesis and modification.

Lipid A from individual transposon mutants was extracted using an ammonium hydroxide/isobutyric acid method [31] and subjected to negative ion matrix-assisted laser desorption ionization time-of-flight (MALDI-TOF) mass spectrometry analysis [10]. Briefly, pellets from 5 ml of O/N cultures of individual Fn mutants were resuspended in 400 μl isobutyric acid/1M ammonium hydroxide (5:3, v:v) and incubated at 100 °C for 2 h, with occasional vortexing. Samples were cooled on ice for 5 min and centrifuged (2,000 x *g*) for 15 min at RT. The resulting supernatant was transferred to a new tube, diluted with an equal volume of water, and lyophilized. The dried lipid A was resuspended in 400 μl methanol and centrifuged as above, twice. The lipid A pellet was solubilized in 200 μl chloroform/methanol/water (3:1.5:0.25, v:v:v) and spotted (1 μl) directly onto the MALDI sample plate, followed by 1 μl of 20 mg/ml 5-chloro-2-mercapto-benzothiazole (CMBT) MALDI matrix dissolved in chloroform/methanol (1:1, v/v). All MALDI-TOF experiments were performed using a Bruker Autoflex II MALDI-TOF mass spectrometer (Bruker Daltonics, Incorporated). Each spectrum was an average of 300 shots. A tuning mix (Agilent) was used to calibrate the MALDI-TOF. Spectra obtained from the individual transposon mutants were compared with that of WT Fn. MALDI-TOF results were confirmed by large phenol preparations of LPS/lipid A for specific mutant strains [10].

### In vitro replication and stimulation of mouse alveolar macrophage cell line.

The MH-S mouse alveolar macrophage cell line (ATCC) was maintained at 37 °C with 5% CO_2_ in RPMI 1640 medium supplemented with 2 mM L-Glutamine (Sigma), 10mM HEPES (Invitrogen), 10% fetal calf serum (Hyclone), 100 units/ml penicillin, and 100 μg/ml streptomycin (Sigma). For the intracellular replication and stimulation assays, MH-S cells were washed four times with media without antibiotics and plated in 24-well plates precoated with 0.01% poly-L-lysine (Sigma) at a cell density of 10^5^ cells/well overnight, as previously described [10]. Cells were infected the next day with WT or mutant bacteria at the indicated multiplicity of infection. For intracellular replication experiments, cells were washed with 1x phosphate-buffered saline (PBS, Sigma) four times after 2 h of infection. To harvest bacteria, saponin (0.2% saponin/PBS) was added to each well to a final concentration of 0.1% (w/v). Bacteria were harvested at 24 and 48 h after infection. Bacterial counts were performed by plating serial dilutions of bacteria onto TSBC plates and incubating at 37 °C for 48 h. For stimulation assays, MH-S cells were not washed after infection and culture supernatants were collected from plates 6 and 24 h after infection [10]. TNF-α and MIP-2 were measured in these supernatants using ELISA Duoset kits according to manufacturer's instructions (R&D Systems).

### Virulence and protection studies.

For subcutaneous infection, mice were injected with WT Fn or mutant bacteria into the subcutaneous tissue overlying the abdomen. For pulmonary infection, mice were exposed to aerosolized bacteria using a nose-only inhalation system, as previously described [10]. Bacterial deposition in the lungs in each experiment was determined by quantitative culture of homogenized lung tissue harvested from mice killed immediately after exposure. Mice were observed daily for clinical manifestations of disease. To study development of protective immunity, mice previously infected with mutants were challenged with WT bacteria at 30–35 d post-initial infection and survival of the mice was recorded.

### Bacterial burden in mouse organs.

For subcutaneous infection experiments, mice were infected at the indicated dose. At days 1, 2, 4, 7, 10, and 14 post-infection, mice (*n* = 3 each group) were euthanized by CO_2_ narcosis. Spleen, left lobe of liver, and left lung were harvested and homogenized in 0.5 ml of PBS containing 0.1% saponin. Bacterial suspensions were serially diluted and plated onto TSBC agar plates for quantitative culture. Colonies were counted after incubation at 37 °C for 48 h.

For pulmonary infection experiments, mice were exposed to aerosolized bacteria. Immediately and at 1, 3, 7, and 14 d post-infection, mice were euthanized with an intraperitoneal injection of pentobarbital then exsanguinated by cardiac puncture. Initial bacterial deposition was determined by quantitative culture of homogenized lung tissue harvested from four mice immediately after infection. At the indicated time points, the left lungs, livers, and spleens from four mice in each group were homogenized in 1 ml PBS for quantitative culture.

### Bronchoalveolar lavage cell counts.

At 4, 24, and 48 h after exposure to aerosolized bacteria, mice were euthanized with an intraperitoneal injection of pentobarbital then exsanguinated by cardiac puncture. The trachea of each mouse was cannulated, and the right lung lavaged with 4 0.5-ml aliquots of 0.9% NaCl supplemented with 0.6 mM EDTA [32]. Cell counts in bronchoalveolar lavage (BAL) specimens were measured in a hemocytometer. Differentials were determined from examination of cytocentrifuge slides (Thermo Shandon) that were stained with a modified Wright-Giemsa technique (Diff-Quik; Dade Behring).

### Bone marrow–derived macrophages.

Bone marrow cells were harvested from both femurs and tibias of wild-type C57BL/6, TLR4^−/−^, TLR2/4^−/−^, and MyD88^−/−^ mice. Bone marrow cells were washed and resuspended in RPMI 1640 medium supplemented with 10% FCS, 100 units/ml penicillin, 100 μg/ml streptomycin, and 20% L929-conditioned medium. Cells were grown on bacterial culture plates. Culture medium was added on day 2 and cells were collected on day 5. BMMø were lifted from plates, washed with 1xPBS, resuspended in antibiotic-free medium, and plated into 24-well tissue culture plates. BMMø were infected with bacteria at MOI 1, 10, and 100 and culture supernatants were collected at 6 and 24 h after infection. IL-6 and MIP-2 were assayed using ELISA Duoset kits (R&D Systems). *E. coli* O111:B4 LPS (1000 ng/ml) was used in these studies as a positive control for TLR4/MyD88 activation.

### Data analysis.

Data are expressed as means ± SE. One-way analysis of variance with Tukey's post hoc test or Student *t*-test were used for statistical analysis [32]. A *p-*value of <0.05 was considered significant.

## Supporting Information

Figure S1 Phylogenetic Relatedness of PmrF and PmrK Enzymes among Different Gram-Negative Bacteria (A) Percentage of amino acid homology of FlmF and FlmK proteins. Phylogenetic trees of (B) FlmF and (C) FlmK proteins from Francisella tularensis subspecies *novicida* (U112), *tularensis* (Schu4), and *holarctica* (LVS), Salmonella typhimurium (LT2), Escherichia coli (O157:H7), Yersinia pestis (CO92), and Burkholderia pseudomallei (K96243). Data were analyzed using Geneious software (http://www.geneious.com/). FTN1403 (U112 FlmF1), FTN0545 (U112 FlmF2), FTN0546 (U112FlmK), FTT1433 (Schu4 FlmF1), FTT0454 (Schu4 FlmF2), FTT0455 (Schu4 FlmK), FTL0625 (LVS FlmF1), FTL1611 (LVS FlmF2), FTL1609 (LVS FlmK), STM2298 (LT2 PmrF), STM2301 (LT2 PmrK), YPO2421 (CO92 PmrF), YPO2418 (CO92 PmrK), BPSL1471 (K96243 PmrF), BPLS1474 (K96243 PmrK), ECs3142 (O157:H7 PmrF), and ECs3145 (O157:H7 PmrK).
(159 KB TIF)

Figure S2 Intact In Vitro and Intracellular Replication of Fn Lipid A Mutants Similar growth rate of WT and mutant Francisella novicida in (A) the rich culture medium TSBC at 37 °C and (B) in the MH-S cell line. For the in vitro growth experiment, overnight cultures of WT, *flmF*1, *flmF*2, and *flmK* mutant bacteria were 1:100 back diluted into TSBC and grown at 37 °C. At the indicated time points, 1 ml of culture was sampled for OD_600_ measurement.
(415 KB TIF)

Figure S3 No Hypersensitivity to Antimicrobial Peptide of Fn Lipid A Mutants Disc diffusion assay for sensitivity of WT and mutant Francisella novicida to antimicrobial peptide (polymyxin B) and kanamycin. Log-phase cultures of WT, *flmF*1, *flmF*2, and *flmK* mutant bacteria were plated onto TSBC plates. Polymyxin B (20 μg) or kanamycin (20 μg) were spotted onto blank paper discs (6 mm in diameter) and placed onto the plates. Plates were incubated at 37 °C for 24–48 h before reading the diameter of the clearing zone (clearing zone marked by black rings). Similar to WT Fn, *flmF*1, *flmF*2, and *flmK* mutant bacteria were not hypersensitive to polymyxin B.
(2.8 MB TIF)

## Abbreviations

BMMø: bone marrow–derived macrophages
Flm: francisella lipid A modification
Fn: F. novicida
Ft: F. tularensis
LPS: lipopolysaccharide
MALDI-TOF MS: matrix-assisted laser desorption ionization time-of-flight mass spectrometry
MOI: multiplicity of infection
WT: wild-type
